# The Evolution of Anticancer 3D In Vitro Models: The Potential Role of Machine Learning and AI in the Next Generation of Animal-Free Experiments

**DOI:** 10.3390/cancers17040700

**Published:** 2025-02-19

**Authors:** Carolina Momoli, Beatrice Costa, Lorenzo Lenti, Matilde Tubertini, Marco Daniele Parenti, Elisa Martella, Greta Varchi, Claudia Ferroni

**Affiliations:** Institute for the Organic Synthesis and Photoreactivity—Italian National Research Council, 40129 Bologna, Italy; carolina.momoli@isof.cnr.it (C.M.); beatrice.costa@isof.cnr.it (B.C.); lorenzo.lenti@isof.cnr.it (L.L.); matilde.tubertini@isof.cnr.it (M.T.); marcodaniele.parenti@isof.cnr.it (M.D.P.); claudia.ferroni@isof.cnr.it (C.F.)

**Keywords:** three-dimensional cancer models, spheroids, organoids, tumor-on-a-chip, machine learning, artificial intelligence

## Abstract

The development of anticancer therapies has increasingly relied on advanced 3D in vitro models, which more accurately mimic the tumor microenvironment compared to traditional 2D cultures. This review describes the evolution of these 3D models, highlighting significant advancements and their impact on cancer research. We discuss the integration of machine learning (ML) and artificial intelligence (AI) in enhancing the predictive power and efficiency of these models, potentially reducing the dependence on animal testing. ML and AI offer innovative approaches for analyzing complex data, optimizing experimental conditions, and predicting therapeutic outcomes with higher accuracy. By leveraging these technologies, the next generation of 3D in vitro models could revolutionize anticancer drug development, offering effective alternatives to animal experiments.

## Simple Summary

This review explores the evolution of advanced 3D in vitro models, which better replicate the tumor microenvironment than traditional 2D cultures. It highlights significant advancements in these models and their role in cancer research. The integration of machine learning (ML) and artificial intelligence (AI) is discussed as a way to enhance predictive accuracy, analyze complex data, and optimize experimental conditions. These technologies can reduce reliance on animal testing and improve therapeutic outcome predictions. Together, ML, AI, and 3D models have the potential to revolutionize anticancer drug development.

## 1. Introduction

Despite significant advancements in early detection and targeted treatments, cancer remains a leading global health burden and cause of death [1]. The current challenge in oncology is shifting from traditional “one-size-fits-all” treatments to personalized “one dose–one patient” approaches [2]. Tumors have been shown to be complex ecosystems, consisting not only of cancer cells but also of a variety of host cells, soluble factors, and altered extracellular matrix components, collectively known as the tumor microenvironment (TME). The dynamic crosstalk between cancer cells and the TME significantly influences cancer initiation, progression, metastasis progression, and drug resistance, making it a key therapeutic target and a crucial asset in terms of treatment development [3]. Notably, clinical diagnoses primarily rely on tumor biopsies, which often fail to capture the full extent of intratumoral heterogeneity and may overlook newly emerging, highly aggressive tumor clones. Furthermore, patients with the same cancer subtype can exhibit distinct tumor phenotypes that evolve dynamically during disease progression and treatment, leading to highly variable therapeutic responses, including both natural and acquired resistance to therapy [4].

**Figure 1:**
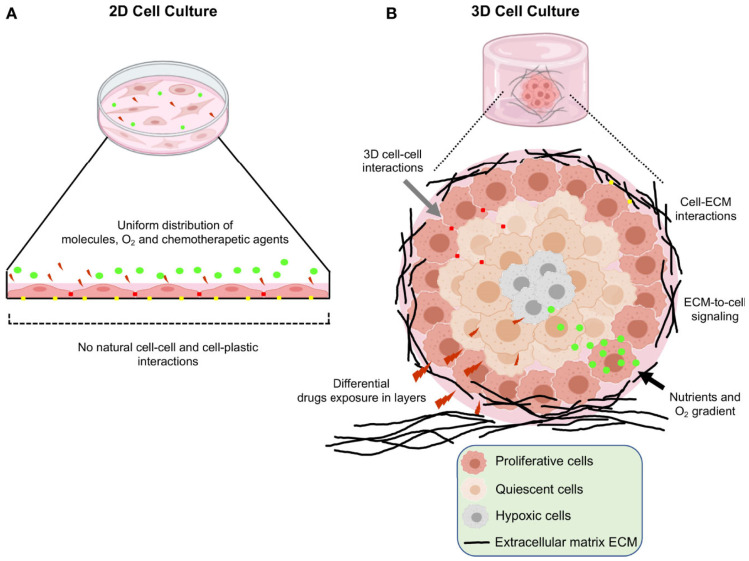
Illustration depicting the key distinctions between 2D and 3D cell cultures. (**A**) Conventional 2D cell cultures, where cells grow in a flattened monolayer on the surface of plastic plates. These cultures are characterized by reduced cell–cell interactions and unlimited exposure to nutrients, oxygen, and drugs, which are significant limitations. (**B**) 3D cell culture systems, which enhance cell–cell and cell–extracellular matrix interactions. These systems have restricted access to nutrients and oxygen, along with heterogeneous drug interactions, allowing for a more accurate mimicry of the tumor microenvironment observed in vivo. Reproduced with permission from [15].

Given their cost effectiveness, availability, high-throughput, easy replication and results’ interpretation, two-dimensional (2D) cell cultures, or monolayers, are commonly used in cellular biology to study diseases and for drug screening. However, a large body of evidence indicates that 2D in vitro cell cultures have various limitations (Figure 1A). First, isolating and maintaining cancer cell lines from patient biopsies can be challenging and often inefficient. Second, once cultured, these cells adhere to and grow on flat synthetic surfaces, losing their original morphology and polarization, which may disrupt critical cellular signaling pathways or alter their responses to external stimuli. Third, cells in 2D cultures often undergo extensive clonal selection, leading to the establishment of derived cell lines that no longer reflect the genetic heterogeneity of the original tumors. Additionally, in vitro cancer cell lines are rarely paired with a patient-matched 2D normal tissue counterpart. Most critically, they fail to replicate the complex network of dynamic interactions present in the three-dimensional TME of living patient tumors, which can significantly influence the effectiveness of cancer therapies [5].

The approach to research in the 21st century is experiencing a significant shift, particularly in how animal models are used as a distinctive method for analysis and investigation. It has been several decades since the three Rs principles (replacement, reduction and refinement) were introduced by Russell and Burch in 1959 [6].

First, researchers should make every effort to substitute animal models with alternative non-animal methods whenever possible (replacement). In the second step, researchers should aim to minimize (reduction) the number of subjects used in an experimental protocol. This can be achieved, for instance, by employing suitable statistical techniques that help determine the minimum number of animals required for a specific experimental design, ensuring that the results are statistically significant with the chosen statistical test [7]. Finally, refinement comes into play when full replacement methods are not feasible, and all strategies in theory and practice have been exhausted to minimize the number of animals used in each experiment. Russell and Burch defined refinement as “any decrease in the incidence or severity of inhumane procedures applied to those animals which still are to be used” [6]. This entirely new approach to designing experiments has prompted the scientific community to explore new and alternative avenues for advancing science in the future [8].

The use of animal models in cancer research aids in comprehending the genetic foundations of cancer, the functions of genes and mutations in cancer formation and contributes to the advancement and evaluation of anticancer drugs [9]. As cancer research advances, an increasing number of animals are being utilized in the creation of animal models. Currently, most of the animal models utilized in cancer research are small animals like mice, rats, zebrafish, fruit flies, and other similar species. Although animal models have been extensively used in cancer-drug development, the low success rates in translating preclinical findings from animals to effective clinical treatments have raised growing concerns about their reliability as predictors of human responses [10,11].

Despite the continued necessity of animal models for progressing to clinical phases, their inherent limitations underscore the need for alternative validation approaches, such as physiologically relevant human in vitro models to study cancer biology and advance therapeutic development [8].

In this view, there is a growing focus on 3D in vitro systems, better mimicking the structure and function of tumors in vivo (Figure 1B). These 3D cultures are gaining prominence in cancer research, serving three key purposes: (i) studying the pathophysiology of cancer progression and resistance [12]; (ii) conducting in vitro screening of anticancer therapies [13]; and (iii) replicating the unique characteristics of an individual patient’s tumors in vitro, enabling personalized screening for the most effective treatments [14].

The 3D models available for validating anticancer therapies are numerous and vary in complexity and application. Tumor spheroids are simple 3D cell aggregates, useful for studying cell–cell and cell–matrix interactions, but limited by their static nature and inability to replicate immune response, fibroblast presence, or vascularization. Organoids are more complex, mimicking tumor architecture but still have similar limitations. To overcome these, tumor-on-a-chip (ToC) models have been developed, incorporating microfluidic technology to better simulate the TME. ToC models offer advantages like precise control of conditions, compatibility with analytical techniques, and faster experimental timelines.

The introduction of these models into industrial high-throughput drug screening, where thousands of compounds are tested at the same time, launch the challenge to establish an accurate, automated system for their analysis providing key information on their potential optimization [16].

In this perspective, the incorporation of artificial intelligence (AI) has the potential to provide huge improvements in terms of predictive accuracy, data integration, model optimization, and the ability to analyze complex datasets, ultimately enhancing the design, personalization, and effectiveness of 3D models for anticancer therapies. AI refers to systems programmed to perform tasks that typically require human abilities, such as learning and problem-solving. Machine learning (ML), a subset of AI, involves developing algorithms that analyze data to identify patterns of behavior [17,18]. ML models are particularly effective when the analyzed system is highly complex and presents numerous variables with hidden relationships between them, including the complex and multifaceted disorder characterized by thousands of genetic and epigenetic variations typical of the tumor [19].

Building on these preliminary considerations, this review aims to provide an overview of the latest applications of 3D models in the cancer therapy discovery pathway, with a particular focus on their integration with artificial intelligence. We will highlight how these models have the potential to revolutionize the preclinical testing landscape by offering more accurate, human-relevant data compared to traditional 2D cultures or animal models. The synergistic combination of 3D models and AI could drive a transformative shift toward more efficient, targeted, and patient-specific cancer therapies, bridging gaps in drug efficacy prediction and personalized treatment design.

Although this is not a systematic review, the literature search was conducted using multiple databases, including PubMed, Scopus and Web of Science to ensure a comprehensive coverage of interdisciplinary advancements. A non-exhaustive list of keywords and phrases employed for the search is as follows: “3D cancer models”, “3D bioprinted tumor models”, “organoid-based drug screening”, “tumor-on-a-chip systems”, “patient-derived organoids”, “tumor microenvironment (TME) modeling”, “artificial intelligence in cancer research”, “machine learning for drug discovery”, “deep learning in oncology”, “AI-driven biomarker identification”, “predictive oncology algorithms”, “neural networks for tumor profiling”, “computational drug repurposing”.

These terms were used to identify pivotal studies, emerging trends, and unresolved challenges in the field, with emphasis on publications from 2012 to 2024.

## 2. Three-Dimensional Models

### 2.1. Development and Relevant TME Features

Since the initial discovery of the ability to replicate an in vivo-like environment, the 3D approach has emerged as a groundbreaking innovation in the field of alternative methods. A 3D cell culture is defined as “a cell culture that can mimic a living organ’s organization and microarchitecture” [20].

**Figure 2:**
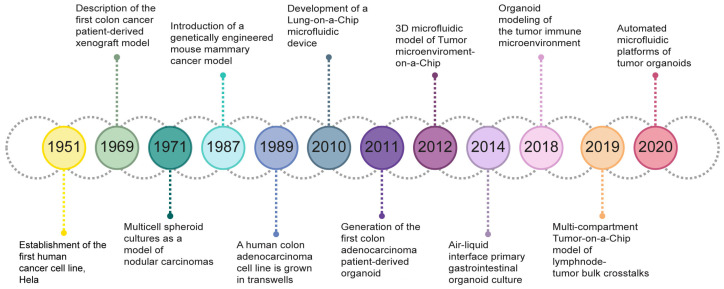
Timeline of milestones in the development of 3D cancer models. Reproduced with permission of [5].

As illustrated in Figure 2, a significant milestone was achieved in 1951 with the isolation of HeLa cells from human uterine cervical cancer tissue. The use of HeLa cells enhanced the adoption of in vitro techniques globally, as they offered consistent and repeatable outcomes due to their immortalized and uniform nature. Spheroids were introduced in the early 1970s by Sutherland and collaborators [21], and since then various models and techniques for their generation have been developed.

In 2010, Huh and colleagues developed the first lung-on-a-chip microfluidic device that reconstituted the critical functional alveolar–capillary interface of the human lung. This bioinspired microdevice mimics complex, integrated organ-level responses to bacteria and inflammatory cytokines introduced into the alveolar space [22].

**Figure 3:**
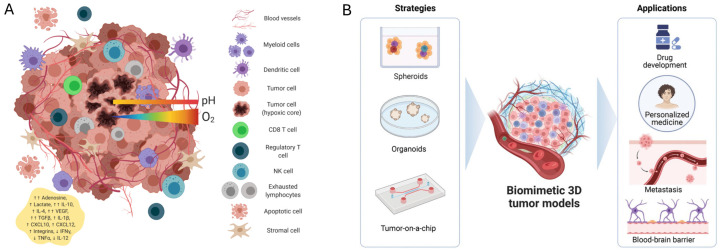
(**A**) The tumor microenvironment (TME). Reproduced with permission of [26]. (**B**) Schematic overview illustrating in vitro strategies for replicating the human TME in three dimensions, including spheroids, organoids, tumor-on-a-chip models and some of their applications. Reproduced with permission of [27].

In recent years, 3D cell models have attracted increasing attention in cancer research due to their ability to closely mimic several hallmarks of in vivo tumors, including the TME. The TME is distinguished by several key characteristics, including low extracellular pH, elevated levels of reactive oxygen species (ROS), hypoxia, and a predominantly immunosuppressive niche compared to normal tissue [23]. As shown in Figure 3, the neoplastic cells are heterogeneous assemblies of infiltrating or resident host non-neoplastic cells—such as T and B lymphocytes, natural killer cells, dendritic cells, monocytes, endothelial cells, pericytes, cancer-associated fibroblasts (CAFs), mesenchymal stromal cells, and adipocytes—alongside niche-specific soluble factors (e.g., cytokines, growth factors, metabolites, enzymes, miRNAs) and a modified extracellular matrix (ECM).

Mounting evidence underscores the critical role of the dynamic interplay between cancer cells and components of the TME in shaping disease progression, including cancer initiation, metastasis, and drug resistance, making the TME a promising therapeutic target. To achieve more durable, side-effect-limited or, ideally, side-effect-free personalized anticancer strategies, it will be essential to accurately model both cancer heterogeneity and the complex interactions within the TME [24].

Hypoxia, a condition of low oxygen and nutrients common in larger solid tumors, significantly impacts cancer progression by upregulating the glucose metabolism, activating angiogenic growth factors and receptors, recruiting pro-tumor immune cells, and tumor-associated macrophages [23]. This hypoxic condition cannot be mimicked in 2D systems but can be recreated in various 3D culture platforms, such as spheroids, organoids and ToC [25].

Numerous initiatives have taken place in recent years to create 3D models that are physiologically relevant and can completely mimic the functioning of tissues and organs (Figure 3B). Overall, 3D cultures present a robust platform for exploring critical tumor processes that mirror in vivo conditions. They offer an alternative to using laboratory animals; indeed, the toxicity levels observed in certain 3D cell cultures closely match those found in animal studies.

The following sections aim to provide a comprehensive and up-to-date overview of spheroids, organoids, and ToC models in the development of cancer treatments, covering the methodologies for creating these 3D models, their respective strengths and limitations, and their contributions to advancing our understanding of tumor biology and improving therapeutic strategies.

### 2.2. Spheroids

**Figure 4:**
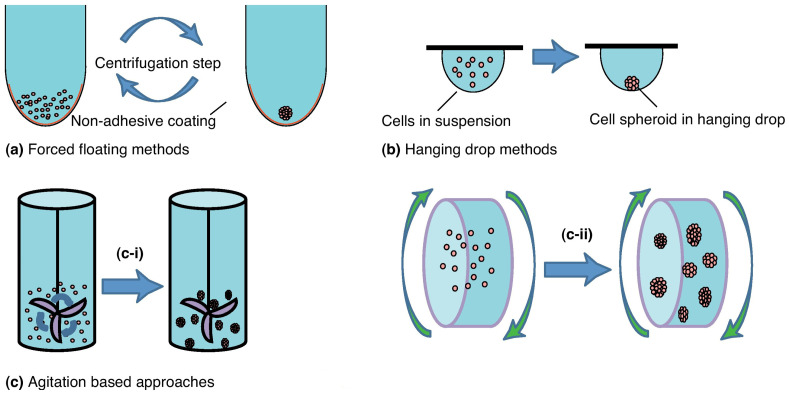
Most used methods to generate multicellular 3D spheroids. (**a**) Forced-floating of cells, (**b**) hanging drop method and (**c**) agitation-based approaches: (**c**-**i**) spinner flask bioreactors and (**c**-**ii**) rotational culture systems. Adapted with permission of [33].

Since their initial introduction in the 70s, various spheroid models and techniques for their generation have been developed. Spheroids, typically grown as free-floating clusters of spherical cell units, are regarded as having a relatively simple structure for modeling tumors [28]. Spheroids mimic key tumor characteristics such as diverse cell types, signaling pathways, growth rates, ECM production as well as its interactions with cells [29], gene expression profiles resembling in vivo settings, and a multi-layered tumor structure [30]. Techniques like hanging drop, agitation-based technique and the liquid overlay techniques can be utilized to create cell spheroids (Figure 4).

The liquid overlay technique (Figure 4a) consists of cells seeded on non-adhesive surfaces to avoid attachment. This method has many benefits: it is simple and generally reproducible as equal numbers of cells can be seeded into each well to produce consistent spheroids. As a result, spheroid sizes can be easily adjusted—larger spheroids can be obtained by seeding a greater number of cells [31]. The primary drawbacks of these methods include the variability in the size of the resulting spheroids, inconsistent cell distribution and composition, along with demanding labor needs.

Mayer and his colleagues developed multicellular gastric cancer spheroids utilizing this technique and they compared them with the corresponding xenografts in immunodeficient mice. They showed that 12 out of 17 gastric cancer cell lines could replicate the characteristics of the original carcinoma when spheroids were cultured utilizing this method [32]. In conclusion, this technique offers a simple, scalable, and cost-effective approach for 3D spheroid culture, suitable for drug screening and other applications. While adaptations to analysis methods are required, this model provides a reliable tool for studying cancer biology and treatment responses.

The hanging drop technique (Figure 4b) is based on the use of a small aliquot (typically 20 µL) of a single cell suspension which is then seeded in the lid of a Petri dish; after seeding, the lid is then turned over and the aliquots of cell suspension turn into hanging drops that are kept in place due to surface tension. A saline solution is added to the bottom of the Petri dish to prevent evaporation of the drops [33]. This method is relatively simple and has been reported to have a reproducibility of almost 100%. Kelm et al. demonstrated that 3D spheroids generated using this technique with HepG2 liver cancer cells and MCF-7 breast cancer cells were patho-physiologically relevant. The resulting 3D structures were highly organized, produced their own ECM, and were therefore described as “tissue-like” [34]. Since this method relies on the natural tendency of cells to adhere to one another, rather than depending on matrices or scaffolds, it eliminates concerns about the potential effects that these external elements may have on the formed 3D structures. A potential drawback of the hanging drop method is the limited volume of the liquid drop containing the cells. Typically, this method can accommodate a maximum volume of 50 µL (including the drug test medium), as the surface tension that holds the liquid on the culture surface is not sufficient to support larger volumes [33]. Another limitation of this approach is related to the culture medium changing; indeed, it is challenging and time-consuming, leading to a restricted production of spheroids [35].

Another method for creating spheroids is the agitation-based technique, as shown in Figure 4c. Here, cells are placed in a container that is continuously stirred to prevent adherence to surfaces, promoting cell–cell interactions instead [33]. As a result, this technique is only suitable for cell lines that can tolerate high shear stress. The primary advantage of this method is its ability to generate uniform-sized 3D tumor models (spheroids) in high yields. The fluid movement within the bioreactor creates a controlled environment that facilitates the transport of nutrients and waste from the spheroid surface, thereby mimicking the transport mechanisms found in vivo [36]. Agitation-based methods for generating 3D spheroids can generally be grouped into two categories: spinner flask bioreactors (Figure 4c-i) and rotational culture systems (Figure 4c-ii). The first category includes a container to hold the cell suspension and a stirring mechanism to ensure the suspension is constantly mixed [37]. Rotating cell culture bioreactors operate similarly to spinner flask bioreactors. However, instead of agitating the cell suspension with a stirring bar or rod, the entire culture container itself is rotated [33]. Brancato et al. recently validated the consistency of a 3D model with in vivo conditions using biodegradable gelatin-based microcarriers in a spinner flask bioreactor [38]. In the proposed system, pancreatic cancer cells were co-cultured with CAFs, leading to the formation of human pancreatic ductal adenocarcinoma microtissues. The cancer cells were found to be the primary proliferating cell population, aligning with observations from other 3D co-culture models. Analysis of ECM gene and protein expression revealed that fibroblasts co-cultured with pancreatic cancer cells transformed into myofibroblasts, expressing desmoplastic markers. This platform highlighted the critical role of stromal–cancer cell interactions in influencing both cell types.

An important characteristic of this 3D model is that tumor spheroids that reach a size over 400 μm can produce oxygen and nutrient gradients similar to in vivo conditions, including a necrotic core, a quiescent area, and a thick outer layer that is actively proliferating [39]. These conditions make them a suitable model for investigating the impact of hypoxia on cancer progression, blood vessel formation, and drug effectiveness. Moreover, larger spheroids mimic the passive perfusion of drug better in a structured stroma.

Tumorspheres, a specific type of spheroid, serve as a floating sphere model to evaluate cancer stem cell (CSC)-related characteristics in vitro. A key feature of these tumor-derived spheroids is their enrichment in CSCs or cells exhibiting stem cell-like properties. Recent models of CSCs propose that cancer cells are hierarchically organized, with CSCs representing a distinct subset that is crucial for cancer propagation due to their capabilities for self-renewal and differentiation, allowing them to regenerate complete cancer structures [40]. In the 1990s, Dick and colleagues discovered CSCs in hematopoietic cancers [41]. Subsequent research indicated that cells with CSC-like properties are also found in solid tumors, including gliomas, colon and breast cancers. This discovery prompted researchers to develop techniques for cultivating CSCs from solid tumors in vitro. Since normal neural cells had already been successfully grown in sphere cultures, it was not surprising that the first successful spheroid cultures of cancer cells involved glioma CSCs [42]. Following these initial studies, tumorspheres were also developed for cancers of the breast, colon, ovary, and prostate [43]. For instance, tumorspheres generated from breast cancer cell lines were enriched in early progenitor/stem cells and had the capacity to differentiate into all three mammary epithelial lineages. The established sphere cells are considered cancer-initiating cells because they express stem-cell markers and have the ability to form xenograft tumors in immunocompromised mice [40].

Another type of tumorspheres is colorectal cancer spheroids (colonspheres); they were initially derived from CD133-positive colon cancer cells cultured in serum-free medium. The resulting sphere cells retained the ability to replicate the histopathological features of the original tumor when implanted in immunocompromised mice [44]. These colonspheres were used to study CSC-related traits, including chemoresistance, metastatic potential, and tumorigenicity at the single-cell level. Additionally, colonspheres were employed to assess the chemosensitivity of novel compounds targeting the Wnt pathway [45].

Although not exhaustive, these examples of tumor spheroids used to study various aspects of cancers highlight the potential of these models to enhance our understanding of the key mechanisms involved in tumor progression and dissemination by providing an environment that better mimics in vivo conditions compared to traditional 2D cultures.

### 2.3. Organoids

**Figure 5:**
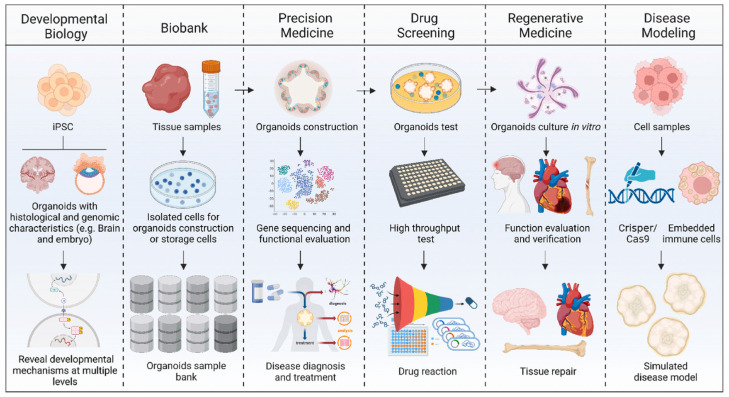
Application of organoids. Reproduced with permission of [49].

While tumor spheroids from cell lines are commonly used to study cancer growth, invasion, and drug screening, they do not accurately mimic the intricate biological and clinical characteristics of primary tumor tissues. This hinders their ability to accurately predict how individual patients will respond to therapy [27]. To address this restriction, organoids have become valuable resources in disease modeling, drug testing, and personalized medicine advancement [46]. The term “organoid” refers to clusters of cells that grow in a defined three-dimensional in vitro environment, where they self-organize and differentiate into functional cell types. These mini-clusters mimic both the structure and function of a natural organ/tumor in vivo, and are sometimes referred to as “mini-organs” [30]. Organoids can be derived from various types of stem cells, including embryonic stem cells, induced pluripotent stem cells (iPSCs), or neonatal/adult stem cells, through a process that mirrors how an organ develops its unique structure [47]. The key point is that tumor organoids retain the essential structural and functional characteristics of real tumors, potentially offering a highly predictive in vitro model for guiding clinical decisions [27]. Their applications range from high-throughput drug screening to sophisticated disease modeling, and some have even progressed to clinical translation (Figure 5). For instance, organoids have been utilized to recreate complex TME by co-culturing with immune cells, significantly enhancing our understanding of cancer biology [48].

In drug discovery, organoids provide platforms to evaluate the efficacy and toxicity of new therapeutic agents. Liver organoids, for example, are valuable tools for studying drug metabolism and identifying novel drug targets [50]. In regenerative medicine, they present the possibility of generating functional tissues for transplantation and other therapeutic purposes [51]. Additionally, organoids are pivotal for exploring human developmental biology, offering critical insights into the mechanisms of various diseases, including genetic and infectious disorders. For example, brain organoids enable the study of human brain development and the underlying causes of neurological disorders [52]. As organoid technology continues to evolve, it promises transformative advancements in these areas, deepening our understanding of human biology and opening the door to innovative therapeutic strategies.

The first example of an organoid was published in 2009 by Sato’s group [53]. They demonstrated how single adult intestinal stem cells expressing leucine-rich repeat-containing G protein-coupled receptor 5 could form 3D intestinal organoids within Matrigel, a gelatinous protein mixture rich in extracellular matrix components. It primarily consists of laminin, collagen IV, entactin, and heparan sulfate proteoglycans, which provide a supportive environment resembling in vivo conditions for cell growth and differentiation. These organoids were able to self-organize and differentiate into crypt-villus structures without requiring a mesenchymal niche. This study was the first to establish a 3D organoid culture from a single adult stem cell, paving the way for further development of organoid models in various systems, including endodermal organs (e.g., stomach, liver, pancreas, lung, and kidney) and neuroectodermal tissues (e.g., brain and retina) [47].

Recently, extensive tumor biobanks have been created through 3D cultivation of patient-derived organoids (PDOs), which preserve the genetic and histological diversity of the original tumor tissues [54]. To date, the PDO culture system has been improved and created for various types of cancer, such as breast, colon, gastric, prostate, pancreatic, and renal carcinoma [55]. Weeber et al. developed PDOs from tumor biopsies of 14 patients with metastatic colorectal cancer. Using SOLiD sequencing (sequencing by oligonucleotide ligation and detection), they analyzed 1977 cancer-related genes within both the PDOs and their respective original tumors. The study found a 90% similarity in somatic mutations between PDOs and the corresponding patient biopsies, highlighting the potential of organoids for genomic-based personalized medicine [13].

In a different study, Van De Wetering et al. created a tumor organoid biobank from 20 successive patients with colorectal carcinoma (CRC). Whole exome sequencing demonstrated that this living biobank accurately reflects the somatic copy number variations and range of genetic alterations present in CRC. This study also showed that these organoids are suitable for high-throughput screening (HTS) and facilitate the identification of gene–drug associations [55]. Various clinical trials across different cancer types have been carried out to assess the potential of tumor organoids in personalized cancer treatment. In the clinical study NCT04736043 (https://clinicaltrials.gov/study/NCT04736043?term=NCT04736043&rank=1; accessed on 6 November 2024) the researchers generate organoids from pancreatic cancer tissue obtained through both endoscopic ultrasound-guided fine needle aspiration and endoscopic ultrasound-guided fine needle biopsy during the diagnostic process as well as from tissue obtained after surgery as part of the treatment process. To assess drug responsiveness, they perform cell viability assays on the organoids that were treated with various anticancer drugs, including those used in adjuvant chemotherapy for pancreatic cancer. Additionally, they conduct genomic analysis on each organoid to identify any unique mutations. By examining the relationship between these genomic mutations and the organoid’s response to anticancer drugs in patients eligible for surgery, the researchers aim to tailor adjuvant chemotherapy strategies post-surgery, ultimately developing a platform to predict individual patient outcomes. The clinical trial, NCT04279509 (https://clinicaltrials.gov/study/NCT04279509?term=NCT04279509&rank=1; accessed on 6 November 2024), is a single-center study designed to prospectively evaluate whether high-throughput drug screening using PDOs can reliably identify chemotherapeutic agents that lead to objective responses in patients with refractory solid tumors, including head and neck squamous cell carcinoma, colorectal cancer, breast cancer, and epithelial ovarian cancer.

Cancer biomedicine aims to understand both the biological distinctions between tumor types and the variations across individual patients, even within the same pathological classifications. Consequently, there has been a growing focus on analyzing the TME, which has led to several promising findings. PDO technology has played a vital role in advancing personalized or precision medicine in cancer research. However, due to its purely epithelial origin, PDOs lack other cell types that comprise the TME, limiting their ability to replicate the structural and physiological components of the entire tumor accurately. To address this limitation, co-cultures of organoids enriched with specific cell types (such as stromal, inflammatory, immune cells, and pathogens) have been developed, creating models that better mimic the complexity of the TME [56].

In gastrointestinal cancer research, organoid co-cultures have proven valuable for uncovering mechanisms in pathogen-induced carcinogenesis [57]. One well-studied example is the chronic infection caused by Helicobacter pylori (H. pylori), which affects about half of the global population and can lead to gastric cancer in some patients [58]. In gastric organoids, H. pylori infection has been shown to increase both cell proliferation and the activation of stem cells via the oncogenic virulence factor CagA [59]. Additionally, organoids infected with H. pylori exhibit inflammatory responses, including NF-kB pathway activation and IL-8 overexpression [60], along with a notable increase in PD-L1 expression [61]. Similarly, salmonella infection has been identified as a genotoxic agent that acts as an “inflammatory stimulus”, potentially contributing to malignant transformation of gallbladder epithelium [62].

Organoid technology represents a breakthrough in biomedical research, but it faces numerous challenges across its construction, data analysis, and application domains. During the construction phase, selecting appropriate matrix materials is critical. Currently, Matrigel dominates the field but presents issues such as poor reproducibility, variability, and potential immunogenicity [63]. The development of synthetic or hybrid hydrogel matrices offers a promising alternative, with advantages like cost-efficiency and closer alignment with the native structure of human organs. However, creating these hydrogels is a complex process due to the diverse chemical compositions and cross-linking mechanisms involved, necessitating iterative optimization [64].

In data analysis, the lack of standardized protocols and real-time monitoring poses significant obstacles. This absence of uniformity introduces variability, which is compounded by manual handling and subjective data interpretation. Furthermore, the limited automation in organoid culture and analysis processes contributes to inconsistencies, undermining the translational potential of organoid technology [65].

The applications of organoids are not without their own challenges, including ethical concerns and economic hurdles. As organoids grow increasingly sophisticated, particularly cerebral organoids, ethical questions about their potential to mimic human consciousness or experience pain become more urgent [66]. Additionally, the high costs of specialized culture media, growth factors, and labor-intensive procedures create barriers to scaling up and clinical translation [67].

In conclusion, while organoid technology provides groundbreaking opportunities to advance our understanding of human biology and disease, it is hindered by challenges in construction, data analysis, and ethical and economic considerations. Addressing these issues systematically is essential to fully harness the potential of this innovative technology.

### 2.4. Tumor-on-a-Chip

Despite both 3D spheroid and organoid models presenting notable advancements compared to 2D cell cultures, there are still unresolved concerns, including physical environmental cues within the TME. Static culture conditions are commonly utilized for spheroids/organoids. However, they are unable to replicate the shear stresses/hydrostatic pressures and interactions between tissues through blood/interstitial flow and vascular perfusion [68]. Therefore, it is crucial to create a reliable evaluation model that can summarize the complex characteristics of the TME and ensure the precise choice of nano-therapeutic agents for patients.

Recent advancements in microfluidic technology and tissue engineering have led to the development of hydrogel-based microfluidic platforms. These platforms are emerging as promising alternatives to traditional experimental models for replicating key TME features and improving the accuracy of patient response predictions. Microfluidic systems are advantageous due to their precise control over microenvironmental variables and the ability to incorporate various cell types in vitro. Notably, these models require significantly fewer cells than patient biopsy samples, enhancing their utility [27]. Additionally, progress in organ-on-a-chip, OoC, techniques has enabled the replication of the micro physiological functions and three-dimensional microstructures of human organs in vivo.

OoC devices are cell cultures created using microfabrication techniques, which merge the benefits of microfluidic technology with 3D cell culture technology to emulate the intricate features of natural organs [69]. Recreating living systems in a lab setting is challenging, so OoCs use “reverse engineering” to isolate the specific functions of target organs [70]. Since their introduction by Prof. Shuler et al., different types of OoC, including liver, lung, heart, kidney, intestine, and multi-organs-on-a-chip, have been created [71]. OoC offers numerous benefits, such as better replication of natural surroundings, ease of use, reduced expenses, and consistency [72]. Cells are grown in tiny chambers inside these small chips, either in 2D layers or 3D suspensions, to mimic organ tissues. The microfluidic parts of OoC platforms replicate the conditions found in living organisms, including flow, pressure, and nutrient levels [73]. Given the increasing prevalence of cancer as a leading cause of death, many researchers are now leveraging OoC technology to test responses to anticancer drugs.

**Figure 6:**
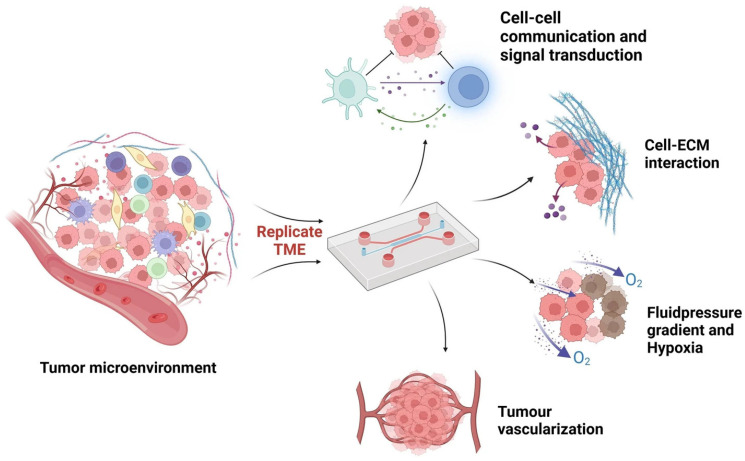
ToC for TME reproduction. Reproduced with permission of [75].

Tumor-on-a-chip (ToC) models are specialized OoC systems that substitute healthy cells with tumor cells to replicate the TME, including the interactions between cancer cells and surrounding healthy tissues (Figure 6). These systems allow for precise control over experimental conditions and enable the study of cancer progression, metastasis, and responses to therapies in a highly controlled setting. ToC models offer numerous advantages that make them highly appealing for both basic and translational research. They enable precise control over cellular, mechanical, and physicochemical conditions, providing a high level of experimental accuracy. Moreover, their compatibility with various analytical techniques, such as transcriptomic analysis and live imaging, significantly deepens the scope of research. ToC models also offer the potential to create human and immunocompetent models, making them highly relevant for personalized medicine. Additionally, the relatively short experimental duration, often just a few days, compared to other 3D cancer models facilitates timely decision-making in clinical settings [74].

Numerous in vitro ToC models derived from human cells have been created to investigate various features related to tumor progression, including cell growth, blood vessel formation, metastasis, and responses to drugs. Fan et al. developed a 3D brain ToC model using polyethylene glycol diacrylate hydrogel as a medium for drug delivery and biological studies. In details, mepitastatin and irinotecan were administered to cells, and this model demonstrated its effectiveness as a glioma chip model for drug screening and release analysis [76]. In another study, Wang and colleagues created a glioma-focused microfluidic device with four parallel chambers to examine the responses of rat C6 glioma cells to the anticancer drug colchicine. Their findings showed notable changes in cell shape and death rate with increasing colchicine doses or extended treatment, supporting the advancement of glioma-targeted anticancer drugs and the development of glial cell-based biosensors for glioma detection [77]. Carvalho et al. designed a colorectal ToC microfluidic device composed of three primary sections. At the center, a circular hydrogel chamber containing ECM and embedded HCT-116 cells includes an inlet and an outlet. Flanking this central chamber are two perfusion channels. Within this setup, CRC cells and human colonic microvascular endothelial cells in the colon respond to the VEGF growth factor. This vascularized colorectal ToC model was created to explore the process of sprout formation and facilitate applications in drug screening [78].

In 2014, Vidi et al. created a breast-on-a-chip platform that simulates mammary cancer ducts by using a monolayer of breast luminal epithelium on a semicircular acrylic base. Tumor cells cultured in these channels exhibit distinct morphologies and drug sensitivities compared to those grown in traditional flat monoculture systems. These results offer valuable insights for the development and testing of cancer therapies [79].

These platforms provide more precise and sensitive responses to therapeutic effects, making them highly suitable for advancing nanodrug delivery system development. In fact, in 2013, Albanese et al. introduced the first spheroid-based model to investigate nanoparticle diffusion in tumors. This model utilized a polydimethylsiloxane microfluidic device to create controlled flow conditions, housing an immobilized multicellular spheroid. The spheroids, composed of MDA-MB-453 breast carcinoma cells, were embedded in an extracellular matrix that anchored them within the microfluidic chamber. This system demonstrated that active targeting of transferrin receptors enhanced nanoparticle accumulation and retention compared to passive targeting with PEGylated nanoparticles [80].

Nowadays, one of the primary challenges in clinical cancer treatment is tumor metastasis. Metastasis accounts for 90% of deaths related to cancer, yet the cellular and molecular mechanisms that drive this process are still not well understood. To address this, numerous microfluidic models of metastasis have been developed to analyze the cellular and molecular components involved in the metastatic cascade, which is essential for identifying new therapeutic opportunities for early intervention [27].

As a result, Skardal’s group showcased the effectiveness of a two-organoid metastasis-on-a-chip platform [81]. Using microfluidics to create circulating flow, this system allowed tumor cells to grow at a primary site, enter circulation, and ultimately settle in downstream liver organoids. In this setup, CRC cells from colon organoids migrated into circulation and later attached to the liver organoids, making it one of the earliest in vitro models to simulate tumor cell metastasis from a three-dimensional primary site to a three-dimensional target site. Recently, the team enhanced the model by expanding the downstream organoids from a single site to four, creating a multisite metastasis-on-a-chip platform to study cancer cells’ metastatic preferences [82].

Microfluidic-based ToC platforms are highly effective for replicating the complexities of the TME but face significant challenges in scalability. The intricate networks of microchannels and chambers that characterize these systems are labor-intensive and time-consuming, making it difficult to scale them for larger experiments or high-throughput drug screening. To overcome these limitations, a multifaceted approach is required. Advanced fabrication techniques such as 3D printing and soft lithography provide efficient and scalable methods for creating detailed microfluidic structures. Additionally, modular design principles enable scalability by allowing smaller, standardized components to be assembled into more extensive and complex systems. By employing these strategies, the scalability challenge of microfluidic-based ToC platforms can be effectively mitigated, unlocking their full potential in cancer research and drug development.

While ToC technology is a powerful tool in cancer research, it has inherent limitations. These models are simplified versions of real tumors, often lacking the complexity and heterogeneity found in human tissues. They may focus on specific aspects of tumor biology, potentially overlooking critical interactions. Notable drawbacks include the absence of immune system representation, challenges in modeling metastasis, and issues related to size restrictions and long-term viability. Ethical considerations, the resource-intensive nature of these systems, and variability between models further hinder their widespread adoption. Additionally, integrating systemic effects and ensuring validation against clinical data remain ongoing challenges.

Despite these limitations, ToC models have greatly enhanced our understanding of cancer. Continued research aims to address these constraints, paving the way for even more effective and reliable applications in cancer biology and drug discovery [19].

## 3. Applications of ML and 3D Models

**Figure 7:**
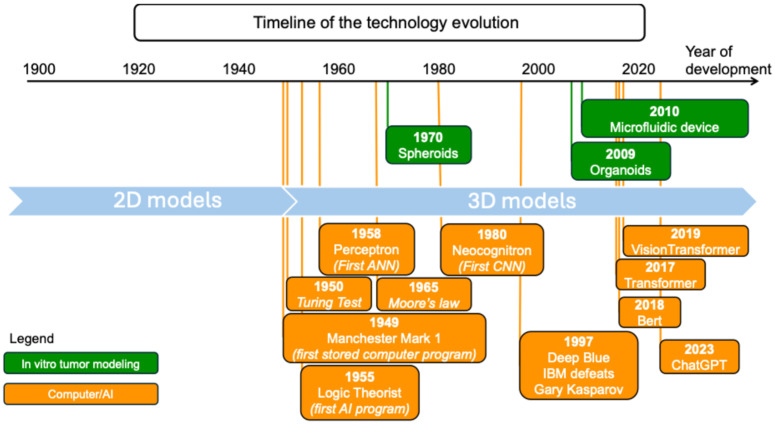
The timeline of the technology evolution for representative techniques used in the context of in vitro tumor modeling and AI. Adapted with permission of [83].

State-of-the-art tumor modeling methods and immune-profiling instruments produce substantial amounts of high-dimensional information. Consequently, in the absence of AI technologies, even advanced tumor-modeling methods and immune-profiling instruments might not empower clinicians and researchers to recreate the TME, anticipate tumor cell behavior, or formulate diagnosis and treatment plans for improved clinical results [83]. Because of this lack, in vitro tumor modeling and AI have progressed significantly throughout the years (Figure 7).

A subcategory of AI is ML that, according to Janiesch et al., is described as “the capacity of systems to learn from problem-specific training data to automate the process of analytical model building and solve associated tasks” [84].

Data used for training can either be labeled or unlabeled before being fed into an ML algorithm, meaning that for a given input, the desired output may or may not be specified. For example, in an image-detection model, the training data can explicitly specify what each image represents, enabling the model to predict the class to which each image belongs. Alternatively, this information can be omitted, and the model will group the data solely based on the input features.

Based on this distinction, ML algorithms can be categorized into three main types: (i) supervised learning, where predictions are made based on prior labeled data; (ii) unsupervised learning, used to uncover hidden patterns using unlabeled data; and (iii) reinforcement learning, which relies on rewards or penalties to guide actions, similar to a video game model [85]. To address some of the latest challenges in healthcare, such as drug development, personalized medicine, and disease diagnosis, ML is being increasingly utilized [86].

A major challenge in this field is the development of anticancer drugs, prompting the use of 3D in vitro tumor models to simulate tumors and their environments for drug testing. By leveraging ML, researchers can reduce the need for extensive in vitro and animal testing, ultimately saving time and costs by performing in silico drug screening or extracting more meaningful insights from fewer experimental tests.

One of the most common methods for analyzing the development of a 3D in vitro model is by acquiring images, typically using various types of microscopies. ML models are then applied to process the images, primarily for two tasks: feature extraction and solving classification or regression problems. Feature extraction is usually performed by convolutional neural networks (CNNs), a type of deep learning (DL) model specifically designed for processing grid-like data such as images [87]. CNNs are particularly well-suited for tasks like image recognition and classification because, according to Benning et al., “CNN takes advantage of the spatial localization inherent in images and hence identifies and extracts features” [88]. This ability to autonomously discover the relevant features makes them especially useful in fields like computer vision, medical image analysis, and video processing [89]. These extracted features can then be used as input for classification or regression models, which can be the same CNN, to predict desired properties—for example, a drug’s effectiveness in killing cancer cells.

An example of AI application is the observation that spheroids undergo characteristic morphological changes when subjected to stress, such as exposure to cytotoxic agents [88]. Images of individual spheroids can then be analyzed and classified using DL models. 

**Figure 8:**
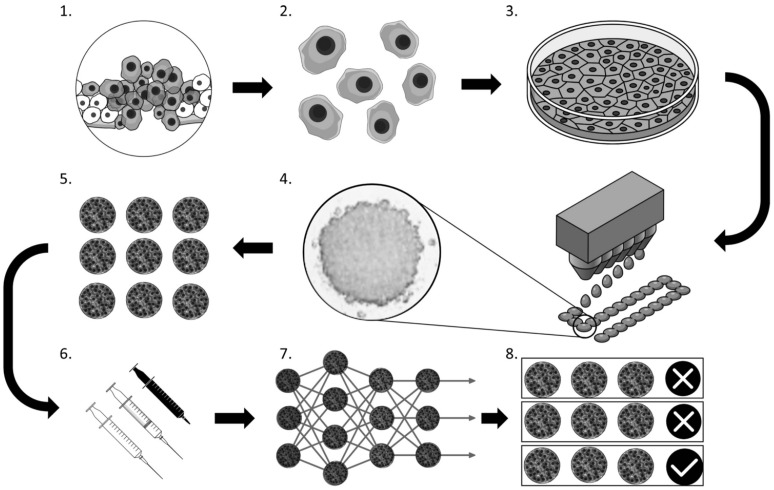
Proposed workflow by Benning et al. to enhance drug screening in solid tumors. (1) A malignant lesion develops in vivo. (2) Primary cells are obtained through biopsy, and (3) cultured to generate a sufficient cell quantity in vitro. (4) Homogeneous spheroids are formed using the hanging drop technique and (5) cultured until they reach full maturation. (6) Various drug combinations and concentrations are administered, and the response of each spheroid is monitored through imaging. (7) The images are processed using a pre-trained CNN, which (8) enables the classification of spheroid sensitivity to specific drugs or concentrations. Reproduced with permission of [88].

In particular, Benning et al. trained a CNN, which takes advantage of the spatial localization inherent in images and hence identifies and extracts features, to estimate the sensitivity of spheroids toward a given drug. This approach can reduce the time- and cost-intensive analysis required to determine spheroid sensitivity to cytotoxic agents, such as flow cytometry, which also demands the production of a large number of spheroids (Figure 8) [88].

A very promising descriptor, that can be extracted by CNNs, is cell motility, which plays a crucial role in life processes and is implicated in various pathologies, such as cancer metastasis [90]. An example of using ML to analyze cell motility in 3D in vitro tumor models is given by Mencattini et al. [91]. They employed a pre-trained DL CNN architecture to extract relevant features from images of cell trajectories, which were acquired with time-lapse microscopy. The sample used consisted of 3D biomimetic gels containing immune cells co-cultured with breast cancer cells in OoC devices, which were treated with an immunotherapy drug. The extracted features were used as input for a classification model, specifically support vector machines to classify trajectories of individual cells based on the presence or absence of the drug.

Another example in the use of CNN is made by Cortesi and colleagues. They introduced ORACLE, a deep-learning neural network designed specifically to identify ovarian cancer cells within complex pre-clinical models [92]. The model was trained on 558 annotated images, featuring both healthy and cancerous cells, which were divided into smaller non-overlapping regions to maximize data input. Each region was labeled with bounding boxes to classify cells as either healthy or cancerous. Leveraging a pre-trained implementation of faster region-based CNNs [93], ORACLE utilizes this widely adopted architecture for object detection. The model’s validation demonstrated impressive performance, achieving an F1 score exceeding 0.9 and a ROC-AUC score of 0.99. This high accuracy, along with its ability to analyze co-cultures of patient-derived cells without the need for fluorescent tagging, marks key advantages of the approach. However, challenges remain, such as the dependency on a high-quality training dataset, which may limit generalizability to diverse biological contexts. Additionally, variations in biological behavior across different cell lines could impact the model’s broader applicability.

Another study by Li et al. presents a breakthrough approach called “deep cytometry”, which uses deep CNNs to classify and sort cells [94]. This method employs time-stretch imaging to capture one-dimensional time-series data from flowing cells. Mode-locked laser pulses convert spatial information into unique waveforms that are recorded by an analog-to-digital converter. By segmenting the waveforms into overlapping portions, the dataset is expanded, enabling the CNN to directly analyze raw data without traditional image processing. The results of this method were outstanding, with an F1 score of 95.71% and accuracy of 95.74% in classifying cancerous and non-cancerous cells within milliseconds. This approach not only improves speed and accuracy but also holds great potential for real-time applications, particularly in label-free early cancer detection.

Oliver et al. developed a 3D microfluidic in vitro device that recapitulates the blood–brain niche in order to understand the development of micro-metastasis and changes to the TME [95]. Fluorescent images of the 3D microfluidic blood–brain niche were captured at different time points using confocal microscopy and reconstructed in three dimensions with confocal tomography. Phenotypic measurements (e.g., cell size, shape descriptors, distance from the endothelial barrier) were extracted from these images. Then, these measurements were used as features for an ML model to predict the metastatic potential of cancer cells. This was used to identify intrinsic phenotypic differences between brain-metastatic and non-brain metastatic cancer cells [96].

A different approach is provided by Yoo et al., exploring the use of spheroid size (SoS) and hypoxic regions (SoH) for efficient drug efficacy evaluation through ML. They trained an ML model to predict changes in SoS and SoH over time using images of untreated spheroids. Then, HepG2 spheroids were treated with sorafenib, a molecule known for its potential to inhibit tumor growth and suppress angiogenesis [97]. The drug’s efficacy was assessed by comparing the experimental changes in SoS and SoH of the treated spheroids with the ML-predicted changes in SoS and SoH based on the untreated spheroids. Experiments revealed that for spheroids treated with sorafenib, cellular growth was inhibited three days post-treatment, and this study demonstrates that SoS and SoH can be accurately predicted by ML models. This approach holds significant promise for biomedical research, by reducing the time, costs and resources associated with preliminary experiments [98].

While image acquisition is a common method to analyze the development of 3D in vitro models, an effective alternative approach is transcriptomic profiling. As demonstrated by Kong et al., organoids can be analyzed by extracting RNA and obtaining gene expression data [99]. Pharmacogenomic data are derived from 3D organoid culture models, including gene expression profiles and drug response information. This latest pharmacogenomic data is analyzed to identify relevant features associated with drug response, particularly by examining pathways that are proximal to drug targets within a protein–protein interaction network. The selected features, which represent the gene expression levels of these identified pathways, are then used to train an ML model. This model learns to associate specific expression patterns with drug responses observed in the organoid models. Once trained, the ML model can be applied to predict drug responses for cancer patients based on their transcriptomic profiles, enabling predictions about the likelihood of a patient responding to a particular treatment.

**Figure 9:**
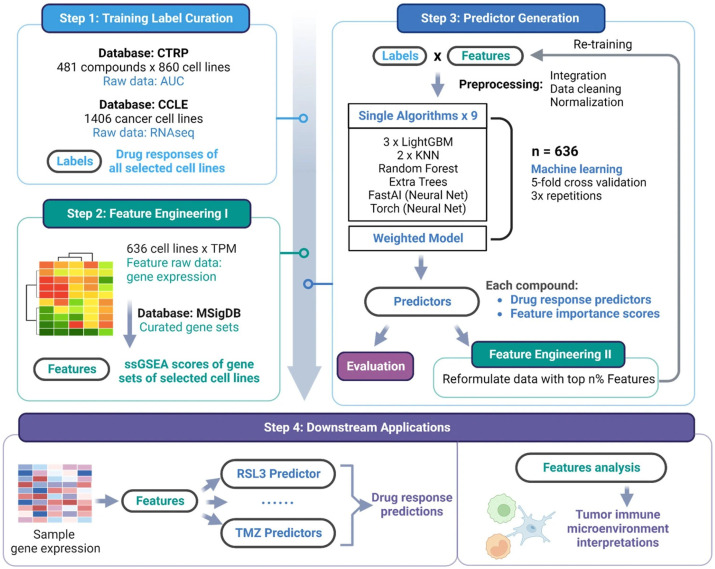
Representation of the four main stages of the GlioML workflow: (1) data preprocessing; (2) feature engineering; (3) training module; and (4) downstream applications, enabling drug response predictions and microenvironment analysis for enhanced therapeutic insights. Reproduced with permission of [100].

Another study by Tang et al. combines 3D bioprinting and ML to enhance the assessment of glioma treatment responses [100]. The authors developed an ML workflow, GlioML, which incorporates nine classic algorithms and a weighted ensemble model for reliable drug response predictions based on gene expression profiles (Figure 9).

These profiles were collected from patient-derived glioma tissue samples and glioblastoma stem cells. The results highlighted the effectiveness of neural networks and gradient-boosting algorithms, which outperformed models like K-nearest neighbors in producing the best predictors. Additionally, the integration of 3D bioprinting enabled the creation of biomimetic tissues that replicated the genomic characteristics and drug responses of native tumors, enhancing the clinical relevance of GlioML’s predictions. However, challenges persist, such as the potential for overfitting when using narrower datasets and the resource-intensive nature of implementing multiple algorithms. Nonetheless, this pioneering approach offers new avenues for improving glioma treatment strategies by incorporating patient-specific drug response insights and addressing the complexities of clinical research.

Sammut et al. presented an ML framework designed to integrate diverse datasets—clinical, digital pathology, genomic, and transcriptomic features—to predict a pathologically complete response in breast cancer patients undergoing neoadjuvant treatment [101]. Using an ensemble learning approach, the researchers developed six predictive models based on varying combinations of features, from clinical data alone to a full integration of DNA, RNA, digital pathology, and treatment data. These data were extracted from the biopsies of 180 women with early and locally advanced breast cancer. The framework demonstrated improved predictive power across multiple cancer types, although challenges remain, such as interpretability and the need for high-quality multi-omic data. Despite these hurdles, integrating clinicopathological and molecular data through ML can significantly enhance predictions of therapy responses, paving the way for more personalized oncology treatments.

In the realm of ovarian cancer, a study from Boehm et al. explored multimodal ML approaches to improve risk stratification for patients with high-grade serous ovarian cancer [102]. By integrating clinical, genomic, histopathological, and radiological data from routine diagnostic procedures, the study developed risk stratification models. These models, which used radiomic features from contrast-enhanced computed tomography and histopathological data from pre-treatment tissue samples, were validated using a late-fusion multimodal statistical framework. The integration of these diverse data types significantly enhanced predictive accuracy. However, challenges emerged in generalizing the models across different institutions, as varying imaging protocols introduced variability. Moreover, reliance on data not originally intended for computational modelling, such as incomplete genomic samples, added complexity. Despite these challenges, the study highlights the potential of integrating quantitative imaging features with clinical and genomic data to improve cancer prognosis. Further exploration of multimodal ML strategies could lead to enhanced cancer prognostics and more effective treatment planning [103,104].

ML models have become transformative tools in drug discovery, enabling the efficient prediction of molecular properties, protein–ligand interactions, and clinical outcomes. These models, particularly DL architectures, excel in processing high-dimensional datasets, extracting complex patterns, and offering predictive insights beyond simple correlative associations [105,106]. Predictive ML models have demonstrated utility in lead identification and optimization, such as predicting absorption, distribution, metabolism, excretion, and toxicity (ADMET) profiles, thus reducing reliance on expensive and time-consuming experimental methods [86].

However, the integration of ML into drug discovery presents several challenges. One critical issue is the dependence on high-quality, domain-specific datasets. Many public and proprietary datasets suffer from noise and inconsistencies, which can lead to biased or overfitted models with limited generalizability [107]. Furthermore, the interpretability of ML predictions remains a significant limitation, especially for models using DL. The “black-box” nature of these approaches can impede the extraction of mechanistic insights, which are crucial for drug design and regulatory approval [18]. Another challenge lies in transferring models trained on existing chemical space to novel or underexplored regions, as these often lack representative training examples. This issue is compounded by the need for rigorous validation strategies, including external validation on independent datasets and experimental confirmation, to ensure clinical relevance. Finally, the ethical and logistical aspects of implementing ML-driven predictions in drug discovery pipelines must also be considered. For example, biases inherent in datasets may inadvertently lead to inequitable drug development or flawed prioritization of candidate molecules [108].

Given the growing complexity of tumor biology, ML applied to 3D tumor models offers a significant advancement over traditional methods, including RNA-based drug response profiling. Unlike transcriptomic snapshots, which provide static molecular readouts, ML approaches in 3D systems capture dynamic cell–cell interactions, migration behaviors, and microenvironmental remodeling, factors that are not discernible through RNA profiling alone. Deep learning models, particularly convolutional neural networks, autonomously extract biologically meaningful features from microscopy images of 3D cultures, offering a scalable and label-free alternative to traditional transcriptomic analyses. Additionally, ML frameworks can integrate RNA sequencing data with imaging, metabolic, and proteomic profiles, allowing for a more comprehensive view of drug response mechanisms that extends beyond differential gene expression analysis. The ability of ML to train on phenotypic datasets further enhances predictive accuracy, enabling the identification of novel drug response markers that might otherwise be overlooked by transcriptomics alone.

## 4. Conclusions and Perspectives

In conclusion, the integration of advanced 3D in vitro tumor models with AI and ML represents a transformative shift in cancer research and drug discovery. These innovative approaches provide unprecedented opportunities to mimic the tumor microenvironment with high precision, enabling better insights into tumor biology, progression, and therapeutic responses. Compared to traditional 2D cultures and animal models, 3D systems such as spheroids, organoids, and ToC platforms offer superior physiological relevance, making them key tools for preclinical testing and personalized medicine.

Looking forward, the combination of 3D models and AI-driven analytics has the potential to revolutionize cancer therapy by facilitating high-throughput drug screening, optimizing treatment regimens, and identifying novel therapeutic targets. However, challenges remain, including the standardization of protocols, scalability of 3D systems, and ensuring robust clinical validation of these models. Future research should focus on addressing these limitations through advancements in synthetic matrices, real-time monitoring technologies, and modular microfluidic designs. Additionally, further integration of AI techniques, such as DL and multi-omics data analysis, will enhance the predictive accuracy and efficiency of these models, paving the way for personalized, patient-specific cancer therapies. By overcoming these challenges, the synergy between 3D in vitro models and AI can redefine the landscape of oncology, offering a path toward more effective and less invasive cancer treatments, ultimately improving patient outcomes and reducing reliance on animal testing.

Therefore, the integration of ML and AI into tumor spheroids, organoids, and OoC models represents a transformative approach in cancer research, offering significant enhancements in predictive power and efficiency. By leveraging ML and AI, researchers can extract more complex, multidimensional data from these 3D culture systems, thereby improving their accuracy in mimicking human tumor biology and reducing reliance on animal testing.

## Data Availability

No new data were created or analyzed in this review paper. Data sharing is not applicable to this article.
