# Correction: Momoli et al. The Evolution of Anticancer 3D In Vitro Models: The Potential Role of Machine Learning and AI in the Next Generation of Animal-Free Experiments. *Cancers* 2025, *17*, 700

**DOI:** 10.3390/cancers17071106

**Published:** 2025-03-26

**Authors:** Carolina Momoli, Beatrice Costa, Lorenzo Lenti, Matilde Tubertini, Marco Daniele Parenti, Elisa Martella, Greta Varchi, Claudia Ferroni

**Affiliations:** Institute for the Organic Synthesis and Photoreactivity—Italian National Research Council, 40129 Bologna, Italy; carolina.momoli@isof.cnr.it (C.M.); beatrice.costa@isof.cnr.it (B.C.); lorenzo.lenti@isof.cnr.it (L.L.); matilde.tubertini@isof.cnr.it (M.T.); marcodaniele.parenti@isof.cnr.it (M.D.P.); claudia.ferroni@isof.cnr.it (C.F.)

## Addition of Authorship

Beatrice Costa was not included as a first co-author in the original publication [1]. The authors state that the scientific conclusions are unaffected. This correction was approved by the Academic Editor. The original publication has also been updated.
